# Pharmacokinetic recall study of Estonian Biobank participants with novel genetic variants in *CYP2C19* and *CYP2D6*

**DOI:** 10.1038/s41525-025-00549-6

**Published:** 2026-01-18

**Authors:** Kristi Krebs, Laura Birgit Luitva, Anette Caroline Kõre, Raul Kokasaar, Maarja Jõeloo, Georgi Hudjashov, Kadri Maal, Elisabet Størset, Birgit Malene Wollmann, Liis Karo-Astover, Krista Fischer, Estonian Biobank Research Team, Volker M. Lauschke, Magnus Ingelman-Sundberg, Espen Molden, Alar Irs, Kersti Oselin, Jana Lass, Lili Milani

**Affiliations:** 1 Estonian Genome Centre, Institute of Genomics, University of Tartu, Tartu, Estonia; 2 Institute of Mathematics and Statistics, University of Tartu, Tartu, Estonia; 3 Tartu University Hospital, Tartu, Estonia; 4 Clinic of Oncology and Haematology, North Estonia Medical Center, Tallinn, Estonia; 5 Center for Psychopharmacology, Diakonhjemmet Hospital, Oslo, Norway; 6 Department of Pharmacy, University of Oslo, Oslo, Norway; 7 Department of Physiology and Pharmacology, Karolinska Institutet, Stockholm, Sweden; 8 Dr Margarete Fischer-Bosch Institute of Clinical Pharmacology, Stuttgart, Germany; 9 University of Tübingen, Tübingen, Germany; 10 Department of Pharmacy, the Second Xiangya Hospital, Central South University, Changsha, China; 11 Institute of Pharmacy, University of Tartu, Tartu, Estonia; 12 Present Address: West Tallinn Central Hospital, Tallinn, Estonia

**Keywords:** Diseases, Drug discovery, Genetics, Medical research

## Abstract

CYP2C19 and CYP2D6 are involved in the hepatic metabolism of approximately 35–40% of clinically used drugs. We conducted an in vivo phenotyping study encompassing 114 Estonian Biobank participants to evaluate the functional impact of rare or novel single-nucleotide and structural variants in the *CYP2C19* and *CYP2D6* genes using omeprazole and metoprolol as respective probe drugs. Plasma concentrations of these drugs and their metabolites were measured at 10 time points, and parent drug-to-metabolite ratios were calculated to determine enzymatic activity. Long-read sequencing enabled high-resolution star allele calling. Our results provide the first in vivo confirmation that partial gene and intragenic deletions in *CYP2C19* (CYP2C19*37 and CYP2C19*42), enriched in Estonians and Finns, are associated with poor metaboliser phenotypes (*P* < 1.2 × 10^−7^). Additionally, we offer in vivo evidence of reduced metabolic activity of the CYP2D6*124 allele and a novel missense variant (c.940C>A) in exon 6 of *CYP2D6*. Furthermore, we observed that inhibitor exposure was significantly associated with higher metabolic ratios for both CYP2C19 (*P* = 3.0 × 10^−6^) and CYP2D6 (*P* = 0.02). Our findings emphasise the importance of identifying genetic variants in *CYP2C19* and *CYP2D6* beyond commonly assessed star alleles and that profiling for drug interactions can provide more precise assignments of metabolic phenotypes and improve personalised treatment.

## Introduction

Cytochrome P450 (CYP) enzymes play a critical role in metabolising many medications. Genetic variants in these enzymes significantly influence drug response across individuals and ethnic groups^1–3^. Among CYP isoforms, CYP2C19 and CYP2D6 are particularly important due to their involvement in the metabolism of widely prescribed drugs, including antidepressants, antipsychotics, proton pump inhibitors, opioids, and cardiovascular agents^2,4–9^. Variants in the corresponding genes account for over 35% of the 60 gene-drug pairs with therapeutic management recommendations supported by FDA data^10^, where 23 relate to *CYP2D6* and eight to *CYP2C19*. ClinPGx^11^ currently lists 19 *CYP2D6*^12^ and 11 *CYP2C19*^13^ gene-drug pairs along with the therapeutic recommendations from the Clinical Pharmacogenetics Implementation Consortium to optimise drug therapy. Genetic polymorphisms in *CYP* genes result in different metabolic phenotypes: poor metabolisers (PM), intermediate metabolisers (IM), normal metabolisers (NM), rapid metabolisers (RM) and ultrarapid metabolisers (UM). PMs have alleles encoding nonfunctional enzymes, while RMs and UMs exhibit increased gene expression or enzyme activity, the latter phenotype caused by gene duplications^14,15^. Activity scores (0 to ≥3.0) are also used to quantify CYP2D6 metabolic capacity^14^.

Advances in sequencing technologies, including long-read and targeted sequencing have highlighted the significance of structural variants (SV) and rare single nucleotide variants (SNV) in pharmacogenetic research, particularly for the *CYP2C19* and *CYP2D6* genes^16–21^. Despite identifying many rare variants, assessing their functional impact remains challenging^22,23^, as computational predictions often fail to accurately determine effects on drug metabolism^24^. While *CYP2D6* deletions and duplications are well-studied^25–28^, accurate phasing of hybrid genes involving the adjacent *CYP2D7* pseudogene in tandem with functional (e.g. CYP2D6*13 + *2) or nonfunctional copies of *CYP2D6* (e.g. CYP2D6*68 + *4) remains difficult with short-read sequencing^29^. For *CYP2C19*, the Pharmacogene Variation Consortium (PharmVar)^30^ catalogues deletion alleles like CYP2C19*36 (complete deletion) and CYP2C19*37 (partial deletions involving upstream regions and at least exon 1)^20,31,32^. However, CYP2C19*37 lacks in vivo phenotypic data and the allele is therefore assigned as ‘no function’ but accompanied by a ‘limited evidence’ label^33^, although the absence of exon 1 makes activity unlikely. Since the deletion is not included among the Association for Molecular Pathology’s minimum set of variants that should be included in clinical PGx genotyping assays^34^, most clinical labs do not test for this allele. This highlights the need for direct assessment of metabolic consequences of novel variants, since clinical implementation of pharmacogenetic testing requires robust evidence of phenotypic impact.

Beyond genetics, non-genetic factors like drug-drug interactions (DDIs), disease states, and lifestyle can significantly influence metabolic phenotypes^35^. For example, in a trial CYP2C19 inhibitors were found to cause phenoconversion in 80% of the participants, shifting some from rapid or normal to intermediate or poor metaboliser status^36^. Therefore, integrating phenotypic assessments and DDI considerations is critical for precise therapeutic decision-making and personalised medicine.

Notably, phenotyping with probe substrates constitutes a reliable method for assessing in vivo CYP enzyme activities and serves as an effective strategy to validate and characterise the functional impact of novel and structural genetic variants^37–39^. For CYP2D6 and CYP2C19, the beta1 receptor antagonist metoprolol and the proton pump inhibitor omeprazole can be used as probe drugs in controlled phenotyping studies^39,40^, respectively, as both exhibit major metabolism via the specific enzyme and good tolerability with a low risk of serious adverse drug reactions.

In this study, we recruited Estonian Biobank (EstBB) participants having novel and uncharacterised *CYP2C19* and *CYP2D6* variants and conducted in vivo phenotyping with metoprolol and omeprazole, respectively, to evaluate the functional consequences of these variants. We found important contributions of novel genetic variants as well as drug-drug interactions, which hold promise to improve the prediction of interindividual differences in drug metabolism and efficacy.

## Results

### Study overview

**Fig. 1:**
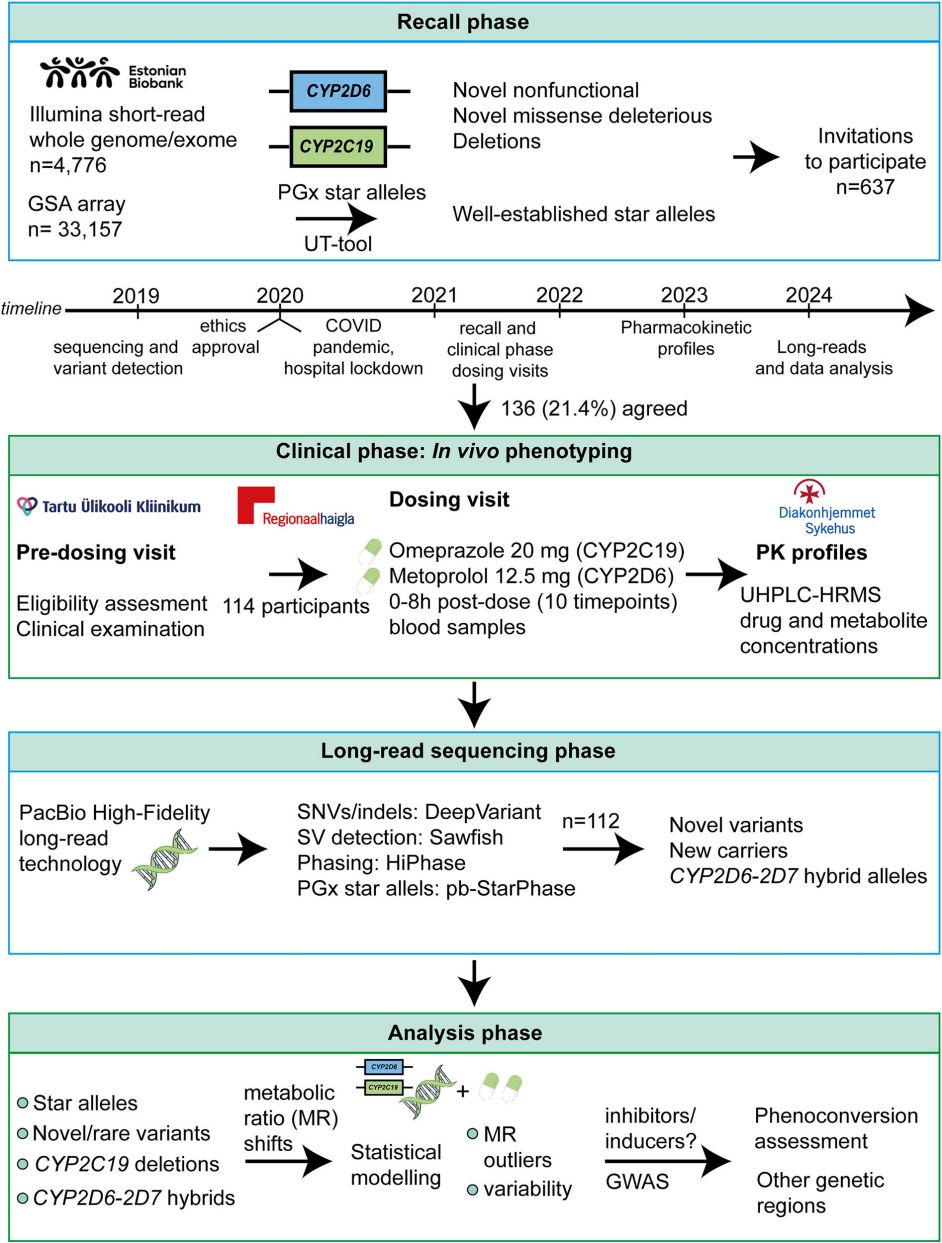
Workflow of the study. Schematic illustration of the different phases of the study, from variant detection and participant recruitment through to the clinical phenotyping and final analysis phases. GS—genome sequencing, PGx—pharmacogenomics, SNV—single nucleotide variant, GWAS—genome-wide association study. *Novel variants* refer to variants without a dbSNP identifier (rsID). *New carriers* refer to additional individuals with recall-eligible variants that were found based on long-read sequencing data. Icons created using Canva.

Of 637 Estonian Biobank (EstBB) participants invited based on genetic inclusion criteria, including rare or novel variants in *CYP2C19* or *CYP2D6* (Supplementary Data 1), and structural variants such as gene deletions (Supplementary Data 2), 174 (27%) consented to participate, and 136 (21%) started the study protocol between 2021 and 2022 (Supplementary Fig. 1). Ultimately, 114 participants (18%) completed all procedures and were included in the final analyses (Fig. 1). The cohort comprised 63.2% women (n = 72) and 36.8% men (*n* = 42), with a mean age of 51 (SD = 13), representing a middle-aged adult cohort (Supplementary Fig. 1). Notably, 50.7% of the women and 47.6% of the men reported taking medications in the 30 days prior to the drug administration visit.

**Fig. 2:**
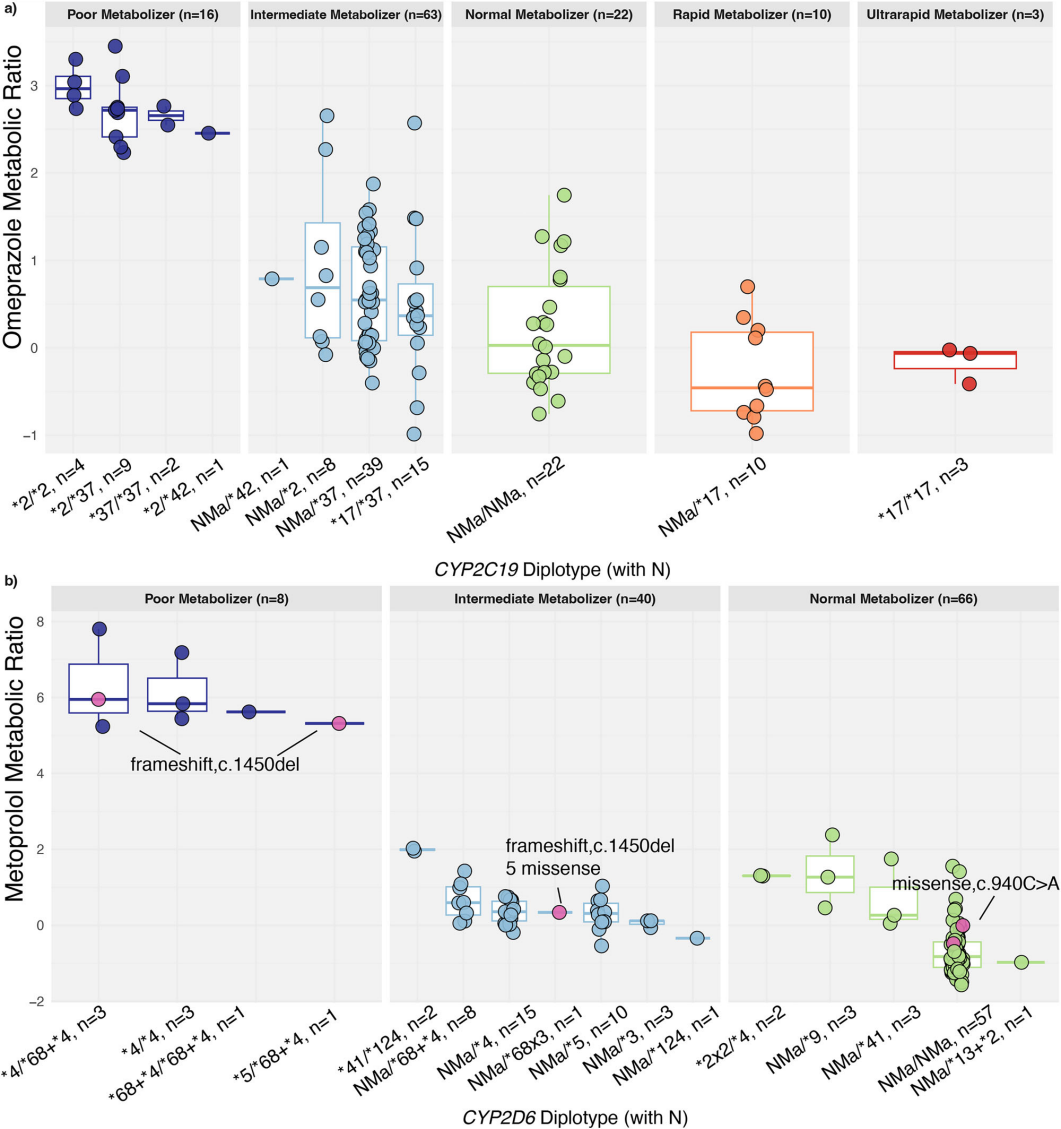
Metabolic ratios across *CYP2C19* and *CYP2D6* star allele diplotypes. The diplotypes (and the number of individuals per group) are given on the *x*-axis, and the log-transformed metabolic ratios of (**a**) omeprazole for CYP2C19 and (**b**) metoprolol for CYP2D6 are plotted on the *y*-axis, respectively. Star alleles with normal function are grouped and denoted as NMa (normal metaboliser alleles); for *CYP2C19*, NMa includes CYP2C19*1 and *38, and for *CYP2D6*, NMa includes CYP2D6*1, *2, *33, and *35. Genotypes are coloured by predicted metaboliser phenotype: dark blue for poor metabolisers, light blue for intermediate metabolisers, green for normal metabolisers, orange for rapid metabolisers, and red for ultrarapid metabolisers. Individuals with missense or nonfunctional *CYP2D6* variants not listed in PharmVar are shown as pink dots. The five missense variants are: c.932C>T, c.911C>T, c.910T>C, c.901G>A, c.899C>G.

Diplotypes were assigned using the pb-StarPhase algorithm, which was considered the gold standard for this study and used for all downstream analyses (Supplementary Data 3, Supplementary Fig. 2). We identified 13 distinct diplotypes for *CYP2C19* and 33 for *CYP2D6* (Supplementary Fig. 2). When normal function alleles were replaced with normal metaboliser alleles (NMa), the counts reduced to 11 diplotypes for *CYP2C19* and 16 for *CYP2D6* (Fig. 2). Additional star allele concordance and comparisons are provided in the Supplementary Information (Supplementary Figs. 3–4, Supplementary Data 4).

### Distribution of predicted metabolic phenotypes for CYP2C19 and CYP2D6

Analysis of predicted metabolic phenotypes based on the determined star alleles revealed that 19.3% of participants (*n* = 22) exhibited normal metaboliser (NM) phenotypes for CYP2C19 while 57.9% of participants (n = 66) did so for CYP2D6 (Supplementary Fig. 5). Poor metaboliser (PM) phenotypes were observed in 14% (n = 16) for CYP2C19 and 7% (n = 8) for CYP2D6. These frequencies are enriched for no functional alleles and do not reflect the general Estonian population. Individuals with PM genotypes demonstrated significantly reduced enzyme activity compared to NM (CYP2C19 β = 2.6, 95% CI: 2.2–3.0, P = 3.5×10^-24^; CYP2D6 β = 6.4, 95% CI: 5.9–7.0, P = 7.1×10^-44^) as shown by increased probe drug metabolic ratios. Compared to individuals with a CYP2D6 activity score of 2.0 (NM), those with a score of 0 showed a significantly increased metabolic ratio (β = 6.6, 95% CI: 6.1–7.0; P = 1.1×10^-50^; Supplementary Fig. 5, Supplementary Data 5).

### Identification of novel *CYP2C19* variants

Long-read sequencing of DNA from 112 participants revealed 595 unique genetic variants in the *CYP2C19* region, including 27 novel variants (not listed in dbSNP) in introns or the 3′ untranslated region (Supplementary Data 6). Some individuals with these variants were phenotypic outliers, showing deviant metabolic ratios, although no firm phenotype-genotype relationship could be identified. None of the individuals with any of the seven novel missense variants initially identified in *CYP2C19* by short-read sequencing participated in the study.

### Functional characterisation of *CYP2C19* deletions

**Fig. 3:**
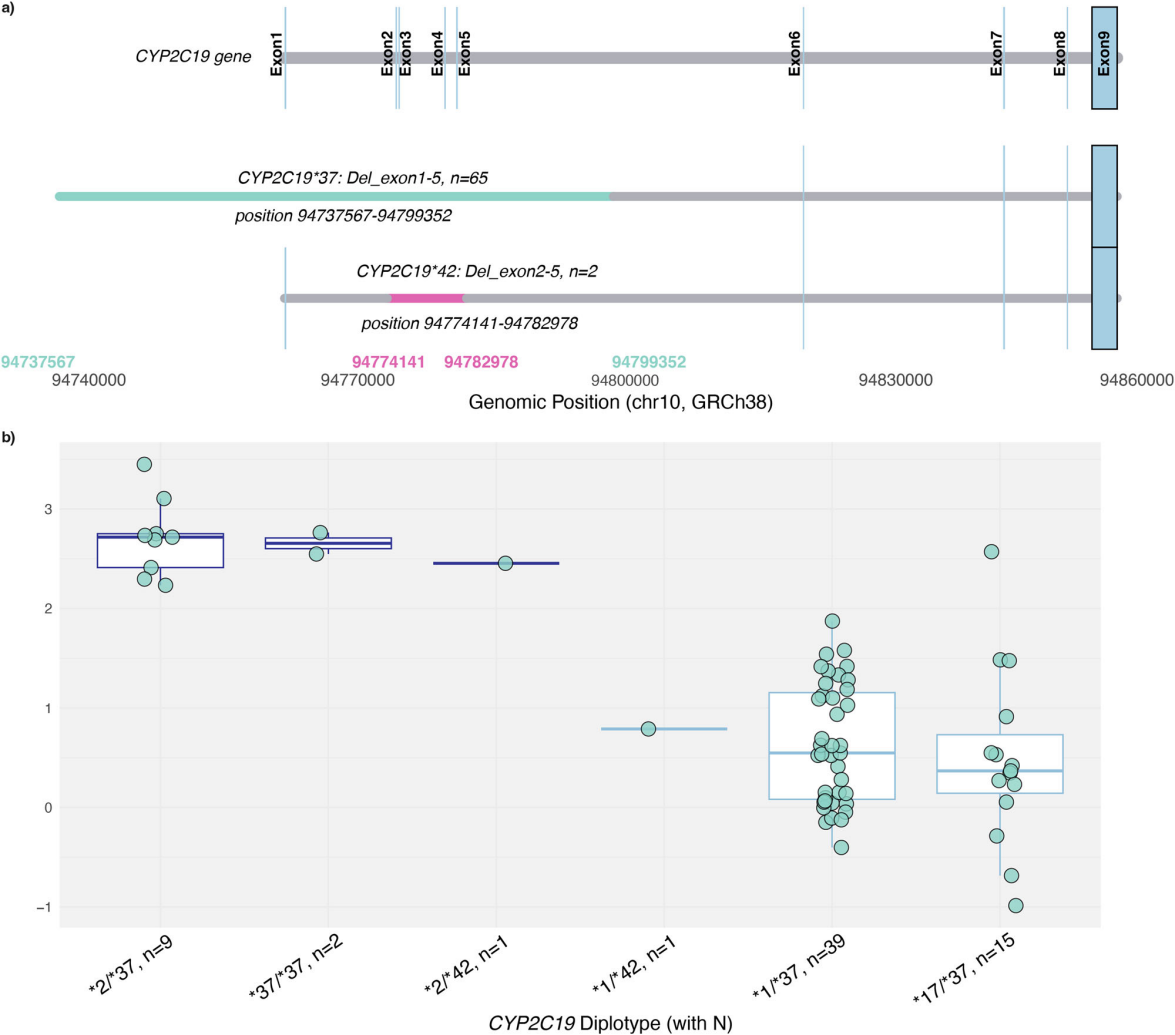
Functional characterisation of CYP2C19 partial deletions and their impact on omeprazole metabolism. **a** Two distinct *CYP2C19* deletions of differing lengths (CYP2C19*37: del_exon1-5 and CYP2C19*42: del_exon2-5) were identified in 67 individuals. The schematic illustrates the *CYP2C19* gene (grey) with exons highlighted in blue (coordinates from Ensembl) and the genomic locations of the two deletions. The CYP2C19*37 deletion extends upstream of exon 1 into the intergenic region between *CYP2C18* and *CYP2C19*, whereas the CYP2C19*42 deletion is an intragenic deletion retaining exon 1. Deletion intervals are indicated in light blue (del_exon1-5) and pink (del_exon2-5). **b** The plot shows the omeprazole log-transformed metabolic ratio in individuals with these deletions.

One of the objectives of this study was to characterise the functional consequences of the relatively understudied CYP2C19*37 allele, which is defined as a partial gene deletion that includes at least exon 1 (Fig. 3). The overall frequency of *CYP2C19* deletions in EstBB was 1.9% based on genotyping array data (Supplementary Information). In our recall study, 65 participants had at least one copy of the partial CYP2C19*37 deletion allele (chr10:94,737,567–94,799,352, GRCh38), and two participants had an intragenic deletion encompassing exon 2 to exon 5 (chr10:94,774,141- 94,782,978, GRCh38). Based on long-read sequencing, all CYP2C19*37 alleles shared identical breakpoints, confirming that they represent the same recurrent deletion event, whereas the intragenic exon 2–5 deletion was unique to the two individuals and was submitted to PharmVar for review. Since the CYP2C19*37 allele is defined as a partial deletion spanning over exon 1, the exon 2-5 deletion was designated a new star allele - CYP2C19*42. Among these 67 participants, two individuals were homozygous for partial deletions (CYP2C19*37/*37) and exhibited a clear PM phenotype when compared to CYP2C19*1/*1 (β = 2.5, 95% CI = 1.7–3.4, *P* = 1.2 × 10^−7^, Supplementary Data 7), consistent with the phenotype typically observed in individuals with the nonfunctional CYP2C19*2/*2 diplotype (no significant difference in metabolic ratio, P = 0.498). When CYP2C19*37 or CYP2C19*42 was paired with a fully functional allele (e.g., CYP2C19*1 or *38), participants typically exhibited an IM phenotype (β = 0.41, 95% CI = 0.10–0.72, *P* = 0.011). These data provide robust evidence that the CYP2C19*37 allele results in loss of function and suggest that the shorter deletion CYP2C19*42 spanning over exon 2 to exon 5 appears to have a similar effect (Fig. 3).

### Identification of novel *CYP2D6* variants

Long-read sequencing identified 94 variants in *CYP2D6* (Supplementary Data 8), 21 of which were not in PharmVar. Seven of the variants were missense or nonfunctional, of which six (c.940C>A, c.932C>T, c.911C>T, c.910T>C, c.901G>A, c.899C>G) were missense and one frameshift (c.1450del), involving a total of five individuals (Supplementary Data 8). This included one novel missense variant in exon 6 (c.940C>A) found in two individuals (Supplementary Fig. 6), which had previously been detected by short-read sequencing in one individual and was used as a criterion for inviting participants to the study (Supplementary Data 1). The variant is predicted to be deleterious (CADD = 22.8), but conclusive functional data is not available. Based on allele assignment, both individuals with this variant were originally genotyped as CYP2D6*1/*1 (no additional *CYP2D6* variants within the gene region), and though we observed a trend towards an increased metabolic ratio, statistical significance was not reached given the small sample size (β = 0.53, 95% CI = −0.36–1.4, *P* = 0.25, Supplementary Data 9). Three other individuals had a rare frameshift variant (c.1450del, rs200304972, gnomAD MAF_MiddleEastern_ = 0.02%, MAF_EUR_ = 0%). One individual with a CYP2D6*35/*68×3 diplotype had six of these seven variants, including the frameshift (c.1450del), all on the CYP2D6*68 allele within the *CYP2D7*-derived sequence. The two other individuals with this variant (c.1450del) had CYP2D6*4/*68 + *4 and *5/*68 + *4 diplotypes. In all three individuals, the c.1450del frameshift variant was consistently observed on the CYP2D6*68 haplotype background, showing no difference in metabolic ratio (Fig. 2). Other CYP2D6*68 + *4 alleles identified in the cohort did not have this variant. Long-read sequencing confirmed the presence of *CYP2D7* sequence downstream of intron 1 in all CYP2D6*68 hybrids, consistent with the canonical *CYP2D6/CYP2D7* hybrid structure. Given that CYP2D6*68 is a nonfunctional hybrid allele, the c.1450del variant located within its *CYP2D7*-derived exon 9 is unlikely to further alter CYP2D6 enzyme activity.

Three individuals had the rare CYP2D6*124 allele (c.1184_1185del, rs757396767, MAF_EUR_ = 0.01%, Supplementary Fig. 6). Two CYP2D6*124/*41 compound heterozygotes had a significant shift toward the PM phenotype (β = 2.7, 95% CI = 1.9–3.6, P = 1.1 × 10^−8^, Supplementary Data 9), with higher metabolic ratios than CYP2D6 NMa/*41 (*P* = 0.02) but significantly different from PMs with CYP2D6*4/*4 (*P* = 1.2 × 10^−10^), indicating a reduced activity by CYP2D6*124. Visual inspection of the metabolic ratio in the single individual with a CYP2D6 NMa/*124 diplotype also suggests that CYP2D6*124 may not confer a complete loss of function. Individuals with the known CYP2D6*9 (β = 1.8, 95% CI = 1.0–2.5, *P* = 8.9 × 10^−6^) and *41 (β = 1.4, 95% CI = 0.71–2.1, *P* = 1.6 × 10^−4^) alleles showed higher metabolic ratios than NMa, consistent with decreased CYP2D6 activity.

Long-read sequencing also enabled the thorough identification of hybrid alleles of *CYP2D6-2D7*. When comparing CYP2D6 NMa/*68 + *4 to CYP2D6 NMa/*4, no significant difference in metabolic ratio was observed (P = 0.30). In addition, individuals having the hybrid allele CYP2D6*68 + *4 in combination with a CYP2D6*4 allele (CYP2D6*4/*68 + *4) exhibited a PM phenotype (β = 7.1, 95% CI = 6.4–7.8, *P* = 1.4 × 10^−35^), in agreement with previous reports describing CYP2D6*68 as a nonfunctional *CYP2D6/2D7* hybrid allele and providing in vivo evidence that the CYP2D6*68 + *4 configuration results in no enzymatic activity.

### Drug–drug interaction (DDI) assessment

Star allele diplotype assignment for *CYP2D6* and *CYP2C19* identified outliers with metabolic activities inconsistent with their genotypes (Fig. 2). To explore potential drug-drug interactions (DDIs) as a cause, we reviewed the drug prescription histories of the participants for the six months prior to the study. Of the participants, 15 (13%) had purchased known CYP2D6 inhibitors, and eight (7%) CYP2C19 inhibitors (Supplementary Data 10).

**Fig. 4:**
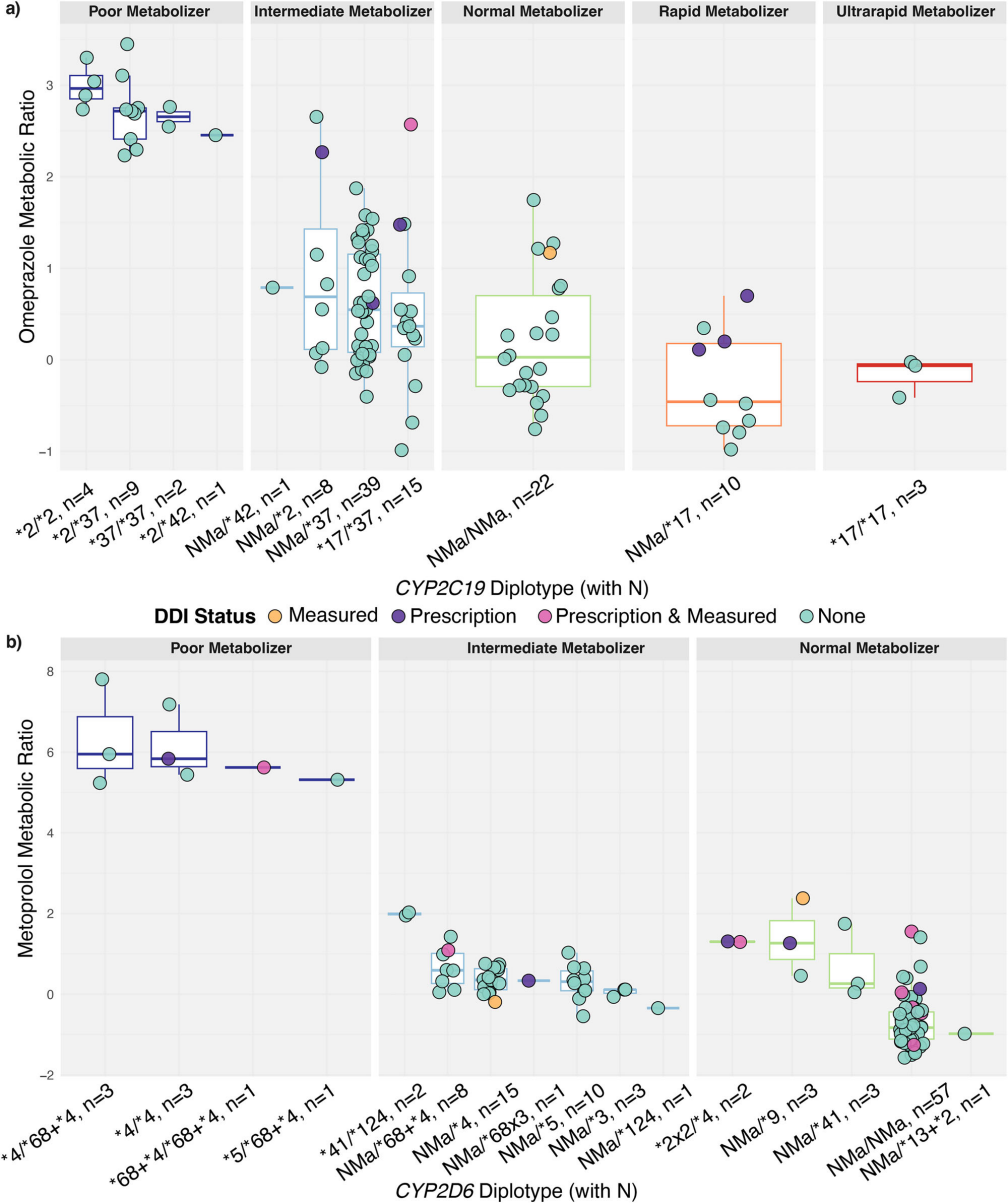
Confirmed presence of interacting drugs and their effect on CYP2C19 and CYP2D6 metabolism: indications of phenoconversion. The log-transformed metabolic ratios are shown for (**a**) omeprazole CYP2C19 and (**b**) metoprolol CYP2D6, stratified by genotype-predicted diplotypes along the x-axis. The number of individuals per diplotype group is indicated in parentheses. Each point represents an individual’s metabolic ratio, with colours indicating drug exposure related to drug–drug interactions (DDI). Blue for no exposure, orange for DDI confirmed via plasma drug detection, purple for DDI inferred from prescription data, and pink for DDIs confirmed by both prescription history and plasma detection. Diplotypes are grouped by predicted metabolic phenotype, with separate facets for Poor, Intermediate, and Normal Metabolisers, and additionally Rapid and Ultrarapid Metabolisers for CYP2C19.

To confirm the use of CYP2D6 and CYP2C19 inhibitors and inducers, we analysed the plasma samples from study participants for the presence of such drugs at the study visit. We detected CYP2D6 inhibitors in 10 (9%) individuals and CYP2C19 inhibitors in two (2%) individuals (Supplementary Data 10). No inducers were found. Notably, some significant metabolic ratio outliers had these inhibitors present, indicating that DDIs likely caused phenoconversion (Fig. 4). For statistical analysis, we included all individuals with detected inhibitors and those with purchases of drugs that could not be tested in plasma (*N*_CYP2C19_ = 8 and *N*_CYP2D6_ = 15, Supplementary Data 10). Interestingly, inhibitor use independently increased metabolic ratio, even after adjusting for diplotype, significant associations for both CYP2C19 (β = 1.1, 95% CI = 0.69–1.6, *P* = 3.0 × 10^−6^) and CYP2D6 (β = 0.49, 95% CI = 0.088–0.90, *P* = 0.02). These findings highlight DDIs as key contributors to phenoconversion in biobank data, and accounting for these effects can substantially improve the analysis of gene–drug interactions.

To other genomic influences on variability in drug metabolism, we performed genome-wide association studies (GWAS) using the metabolic ratios of omeprazole and metoprolol (Supplementary Information, Supplementary Fig. 7, Supplementary Data 11). The results confirmed *CYP2C19* and *CYP2D6* as primary drivers in the metabolism of omeprazole and metoprolol, respectively. This suggests that structural variants, rare SNVs, and DDIs, rather than common variants elsewhere, are the main contributors to the unexplained variability in metabolic phenotypes.

## Discussion

Characterising rare and novel variants in drug-metabolising enzymes is crucial for understanding their phenotypic effects. In this study, we examined the in vivo effect of rare and previously uncharacterised variants in *CYP2C19* and *CYP2D6*, including partial deletions in *CYP2C19* (*37 and *42) that have not been functionally validated yet^20^. Our results highlight the necessity of conducting PK studies with probe drugs to refine pharmacogenetic phenotype assignments and ultimately guide more precise treatment.

Interestingly, the CYP2C19*37 partial gene deletion, reported at a global frequency of ~0.05%^31^, shows higher frequencies in specific populations: 0.1% in European non-Finnish (gnomAD SV v4), 0.43%^32^ to 1.5% (gnomAD SV v4) in Finns, and 1.9% in Estonians (EstBB). Our study confirms that CYP2C19*37 causes loss of enzyme activity as expected due to the lack of at least exon 1 which has the start codon and the membrane integration part of *CYP2C19*. A similar effect was observed for CYP2C19*42. Depending on the genotyping assay, individuals with the CYP2C19*37 allele may be misclassified or return a no-call result, as many routinely tested *CYP2C19* variants are located within the deleted region (exons 1-5). In assays that default missing signals to the reference allele, CYP2C19*37 individuals could be incorrectly reported as CYP2C19*1/*1, potentially leading to inappropriate dosing of essential CYP2C19 substrates such as clopidogrel or certain antidepressants. We also identified a shorter deletion in *CYP2C19* spanning from exon 2 to exon 5. As PharmVar defines CYP2C19*37 as partial deletions where at least exon 1 is deleted, this intragenic deletion does not match the existing CYP2C19*37 definition and represents a distinct partial deletion allele. The allele was submitted to PharmVar for review and it was designated a new star allele – CYP2C19*42. Based on the metabolic ratios observed, individuals with the exon 2-5 deletion showed no apparent differences in enzyme activity compared to those with the exon 1-5 deletion (CYP2C19*37) within the same allelic background. These results emphasise the importance of testing for *CYP2C19* deletions, especially in populations where the frequency of *CYP2C19* deletions is higher than previously recognised, like Estonia^41^ and Finland.

Two participants had a novel *CYP2D6* missense variant (c.940 C > A) predicted to be deleterious in silico (CADD = 22.8). Based on the current star allele definitions, they were genotyped as CYP2D6*1/*1 and would therefore be predicted to have a NM phenotype. However, their metabolic ratios showed a modest reduction in CYP2D6 activity, in line with functional in vitro results, which showed 40% reduced CYP2D6 activity for this variant (p.Leu314Met)^42^. Due to the small sample size, our analysis did not reach statistical significance and further validation in larger cohorts may be necessary for clinical classification of this novel variant.

At the time of recruitment, the nonfunctional *CYP2D6* variant rs757396767 (c.1184_1185del), a two-base pair deletion in exon 8 leading to a frameshift (L395fs), was considered novel and unclassified as part of any star allele. This frameshift mutation now defines the CYP2D6*124 allele^21^. While this allele is predicted to be nonfunctional based on its sequence consequence^43^, this has not been confirmed in any prior in vivo functional studies. Three individuals with this variant participated in our study. The allele sequence corresponded to the canonical CYP2D6*124.001 configuration, with no additional variants detected on the same haplotype. Our findings suggest that CYP2D6*41/*124 compound heterozygotes display reduced metabolic activity compared to CYP2D6*1/*41, thereby supporting a classification of decreased function for CYP2D6*124. However, the CYP2D6*41/*124 diplotype did not result in a poor metaboliser phenotype, further evidenced by a significant difference in metabolic ratio when compared to the known PM phenotype CYP2D6*4/*4. For the one individual with a CYP2D6 NMa/*124 diplotype, the observed metabolic ratio also suggested a partial reduction in function, less than that of other intermediate metaboliser profiles (e.g. CYP2D6*1/*4). In our study, the individuals with CYP2D6*41 (NMa/*41) and CYP2D6*9 (NMa/*9), displayed metabolic ratios at the lower end of the normal metabolizer distribution, similar to those of intermediate metabolizers. This observation aligns with previous reports describing substantial variability in the functional impact of CYP2D6*9 and *41, reflecting the complex and context-dependent nature of decreased function alleles^44^. While these findings provide valuable insights for the CYP2D6*124 allele, the small sample size does not allow us to draw definitive conclusions and a larger number of samples is required to validate these observations.

One of the key findings of this study was the observation of phenoconversion. We confirmed the presence of both CYP2D6 and CYP2C19 inhibitors in the plasma samples from study participants and found a statistically significant association between the presence of CYP2D6 or CYP2C19 inhibitors and metabolic ratios, independent of genotype. Notably, some of the most pronounced outliers in the genotype-metabolic ratio correlations could be explained by the presence of DDIs, highlighting their potential role in explaining discrepancies between genotype and phenotype. Previous studies have underscored the clinical relevance of phenoconversion^35,36^. For instance, a recent study examined the diet-derived biomarker solanidine to assess CYP2D6 activity in elderly patients and found that each additional CYP2D6 substrate or inhibitor was associated with a 0.53-point reduction in the predicted activity score^45^. This emphasises the vulnerability of polymedicated populations to the effects of DDIs.

This study also highlights the value of long-read sequencing technologies for pharmacogenomics. Although short-read genotyping tools like Cyrius and Aldy achieved high concordance for many *CYP2D6* star allele calls, the long-reads and detection of star alleles with pb-StarPhase^46^ offers enhanced resolution for more complex structural variants, like *CYP2D6-2D7* hybrids and phased additional copies of *CYP2D6*. Notably, we did not observe any differences in the metabolic ratio between CYP2D6 NMa/*68 + *4 and CYP2D6 NMa/*4, suggesting that failure to detect the hybrid allele may not always affect phenotype assignment. However, in cases where the hybrid gene appears in tandem with a functional allele, this may result in false detection of a duplication of a functional allele, which would alter the determined metabolic phenotype. Only one individual with the CYP2D6*13 + *2 allele (NMa/*13 + *2 diplotype) participated in our study and displayed a normal metabolic ratio. The long-read sequencing also allowed us to identify one individual with three copies of the CYP2D6*68 allele on one allele, which further confirms that the CYP2D6*68 allele does not always appear in tandem with CYP2D6*4. Because of the technical challenges in accurately identifying hybrid alleles, the characterisation of their functional impact, and the supporting evidence has remained limited. Consequently, no strength of evidence has been assigned to these alleles in ClinPGx translation tables thus far^43^. Consistent with CPIC, PharmVar, and previous reports, CYP2D6*68 and the *68 + *4 tandem are nonfunctional. Our long-read data confirm these structures and provide additional in vivo phenotypic support for their nonfunctional status. Long-read technologies, increasingly cost-effective, are vital for characterizing such variants.

Finally, we recognise several limitations of this study. The relatively small number of participants with certain rare or novel variants—or specific combinations of diplotypes—restricts the statistical power to assign definitive functional classifications or to detect subtle differences in metabolic activity across rare diplotype groups. While we did not identify any individuals with the novel haplotype (*1/*1 + *c.940* *C* > *A*) in combination with a nonfunctional allele in our cohort, such data would provide valuable confirmation of its functional classification. Although our study provides valuable initial insights for some observations, larger and more diverse cohorts, ideally including participants from multiple ethnic backgrounds, will be essential to validate and generalise these findings. Increasing cohort sizes would also facilitate more robust subgroup analyses, such as assessing allele effects across specific age groups, sexes, or comorbidity profiles, which were beyond the scope of the current study. Increasing cohort sizes would also enable more robust analyses of allele-specific activity, which may refine phenotype predictions. Recent work on CYP2D6*17 and *29 demonstrated substrate-dependent activity for risperidone metabolism^47^, illustrating the importance of such investigations for accurate genotype-to-phenotype translation. Additionally, while we thoroughly considered the effects of known DDIs by analysing the presence of known inducers or inhibitors in drug purchase data and the plasma samples of the study participants, other factors may have contributed to the variability seen in the metabolic ratios. These factors include participants’ over-the-counter medications, some food supplements, and general clinical characteristics such as inflammation, as well as additional environmental and physiological influences.

In conclusion, this study elucidates the functional consequences of rare and structurally complex variants in *CYP2C19* and *CYP2D6*. We present PK evidence that *CYP2C19* deletions confer a PM phenotype, emphasising the need to test for structural variants, especially in populations where the frequency of CYP2C19*37 is higher than previously recognised. Long-read sequencing further enhances variant detection, critical for complex alleles prevalent in *CYP2D6*. Our study also highlights the importance of DDIs in combination with pharmacogenetic testing. Future research should prioritise larger, diverse PK studies, integrating multi-omic and environmental data, to advance pharmacogenetic precision.

## Methods

### Variant detection and participant selection

Whole-genome and exome sequencing data obtained with Illumina short-read technology (*n* = 4776) were analysed for the participants of EstBB. The method for sequencing, variant calling, and filtering has been detailed in previous publications^48–51^. In this study, we identified all potential nonfunctional and missense variants in the *CYP2C19* and *CYP2D6* genes in the sequencing data, with a specific focus on novel variants. Variants that did not have a dbSNP identifier (rsID) at the time of recall were considered putatively novel.

Variant annotation and functionality predictions were performed using the Ensembl Variant Effect Predictor (version 87) and the LOFTEE plugin for identifying high-confidence nonfunctional variants. For missense variants, functional predictions were further refined using a prediction framework optimised for pharmacogenetic assessments^52^.

We used a previously developed star allele translation tool^50^—referred to in this study as the “UT-tool”—to assign pharmacogenetic star allele diplotypes for EstBB participants based on Illumina Global Screening array genotype data (*n* = 33,157) and Illumina short-read sequencing data of 4,776 individuals. Deletions were called using the PennCNV software^53^. Only samples with a standard deviation of log R ratio <0.3, absolute waviness factor <0.05, and number of copy number variant calls per sample <100 were retained for further analyses.

For recall, participants were invited based on the following selection criteria:

- Having novel nonfunctional variants in *CYP2C19* or *CYP2D6*;
- Having novel missense variants predicted as deleterious;
- Having the *CYP2C19* deletions (CYP2C19*37, CYP2C19*42), including homozygous individuals and compound heterozygotes with different *CYP2C19* star allele diplotypes;
- Having *CYP2D6* gene deletions or duplications;
- Individuals with well-established reference allele, increased or no function alleles of *CYP2C19* (*1, *2, *17) or CYP2D6 (*1, *4).

Eligible participants were adults (age ≥ 18 years, no upper age limit) who signed the informed consent form. Invitations were sent via email by EstBB personnel between 20/10/2021-23/08/2022. The study complied with all relevant ethical regulations including the Declaration of Helsinki.

### Study visits and eligibility assessment

Responding participants (*n* = 174) underwent a pre-screening phone interview to confirm eligibility. Exclusion criteria included: Clinically relevant hepatic or renal dysfunction; anaemia; pregnancy or lactation; recent history (past year) of alcohol or drug abuse (self-report during clinical interview); use of medications known to interfere with probe drug metabolism; allergy or contraindication to omeprazole or metoprolol; body mass index (BMI) < 18 or >45.

Eligible subjects (*n* = 136) were invited for an in-clinic pre-dosing visit at Tartu University Hospital or North Estonia Medical Centre, where inclusion criteria were verified, informed consent obtained, and relevant clinical and laboratory assessments performed. Concomitant medications were reviewed to identify potential drug–drug interactions.

### Probe drug administration and pharmacokinetic (PK) profile detection

During the probe drug dosing and PK sampling visit, eligibility was re-checked according to the study’s inclusion and exclusion criteria. A clinical examination was performed, and for fertile female participants, the absence of pregnancy was confirmed. Participants received a single oral dose of omeprazole 20 mg (commercially available capsule), metoprolol 12.5 mg (extemporaneously prepared capsule by Tartu University Hospital Pharmacy, as no commercial product was available for this dose). Participants were required to fast prior to probe drug administration to standardise PK conditions.

Blood samples (5 mL each) were collected at 10 time points: pre-dose (fasting) and at 0.25, 0.5, 0.75, 1, 2, 3, 4, 6, and 8 hours post-dose. Samples were drawn from an antecubital vein via an indwelling cannula into EDTA tubes, centrifuged at 1500 *g* for 10 minutes at 4 °C, and plasma stored at −80 °C until analysis. The sampling schedule was based on prior cocktail phenotyping studies that showed 8-hour sampling adequately captures PK profiles in both normal and poor metabolisers^38^. The study subjects were recommended to have breakfast after the drawing of the 1 h blood sample. The composition of this meal was not standardised. Vital signs (blood pressure, heart rate) were monitored throughout the 8-hour visit. A follow-up phone call was conducted after one week to record any adverse drug reactions.

Plasma concentrations of probe drugs and their primary metabolites—omeprazole, 5-hydroxyomeprazole, 5-O-desmethylomeprazole; metoprolol, alpha-hydroxymetoprolol, O-demethylmetoprolol—were quantified using ultra high-performance liquid chromatography (UHPLC) and high-resolution mass spectrometry (HRMS). Samples were analysed at Center for Psychopharmacology, Diakonhjemmet Hospital, Oslo in Norway. Briefly, the plasma samples were prepared by protein precipitation in a semiautomated sample preparation procedure using a Microlab Star pipetting robot (Hamilton, Reno, NV, USA) for liquid handling. The LC system was a Vanquish-UHPLC (Thermo Fisher Scientific, Waltham, MA, USA), and chromatographic separation was performed by an XBridge BEH C18-column (2.6 μm, 2.1 ×75 mm; Waters, Milford, MA, USA) using gradient elution at 35°C with a mix of ammonium bicarbonate buffer (pH = 8.1) and methanol (43–60%). The injection volume was 4 µL and total run time was 2.6 min.

Detection used a QExactive Orbitrap mass spectrometer (Thermo Fisher Scientific) operated in positive ionization mode, acquiring full-scan data at a resolution of 70,000 within the 100- to 1500-Da scan range. The compounds were quantified in full-scan acquisition mode, whereas accurate data-dependent MS2 analysis was simultaneously triggered to permit confirmation of their identification. Within-run and between run CVs and accuracies were <7% and 93–106% for all compounds at the low-quality control level (50 nM for metoprolol and metabolites, and 100 nM for omeprazole and metabolites).

### Measures of enzyme activity

Drug and metabolite concentration measurements were imputed as zero if the analyte was not detected and as LOD/2 if the concentration was below the limit of detection (LOD = 0.4 nM for omeprazole and its metabolites; LOD = 0.2 nM for metoprolol and its metabolites). PK analysis was performed using non-compartmental analysis. The area under the curve (AUC) from time zero until the last concentration measurement was determined for each analyte using the log-linear trapezoidal rule (linear-up log-down method) with the R package pkr. The metabolic ratios were computed as the AUC of the parent drug divided by the AUC of its metabolite to reflect enzyme activity.

The two metabolic ratios calculated for reflecting the CYP2C19 enzyme activity—AUC of omeprazole-to-5-hydroxyomeprazole and -5-O-desmethylomeprazole—were highly correlated (Pearson correlation 0.95). Therefore, we are reporting the results using the AUC of omeprazole-to-5-hydroxyomeprazole only. The metabolic ratio using the AUC of metoprolol-to-alpha-hydroxymetoprolol was used for reflecting the CYP2D6 enzyme activity, because the metabolic ratio using O-demethylmetoprolol was less effective in separating the different groups of CYP2D6 metabolisers. The metabolic ratios were log-transformed in all statistical analyses.

### *CYP2C19* deletion frequency in the Estonian Biobank

To estimate the frequency of the *CYP2C19* partial gene deletion (CYP2C19*37 or CYP2C19*42) in EstBB, individuals with deletion were identified using PennCNV^53^ applied to genotyping data from the Illumina Global Screening Array in 17 batches. Duplicates and samples with call-rate <0.95 were excluded. We only considered deletion calls that (i) fell into the boundaries of an established 61.8k deletion overlapping *CYP2C19* exons 1 to 5 (gnomAD structural variants v4.1.0 variant ID: DEL_CHR10_28B50744)^31^, and (ii) were at least 5k base pairs long. All other *CYP2C19*-overlapping deletions were flagged as ambiguous.

### Post-recruitment star allele re-assignment with new tools

Since pharmacogenetic star allele calling tools keep evolving, we performed additional star allele assignments for a subset of participants (*n* = 43) who had short-read whole-genome sequencing data available. We determined star allele diplotypes for *CYP2D6* using two specialised freely available computational tools: Cyrius v1.1.1^54^ and Aldy v4.5^55^, using default parameters and the GRCh38 reference genome in the calling process.

The diplotype results from these tools were compared against two benchmarks: the UT-tool^50^, which was used for star allele calling during the recruitment phase, and pb-StarPhase^46^, which we consider the analytical gold standard in this study due to its integration of long-read sequencing, phasing, and *CYP2D6-D7* specific reference sequences for accurate alignment and star allele calling^46^. The UT-tool reports diplotypes based on the set of defined *CYP2C19* and *CYP2D6* star alleles in the tool database and does not detect or assign novel SNPs or previously uncharacterized haplotypes. For *CYP2C19*, we expanded the allele calling to include all 114 participants by applying the PharmCAT algorithm^56^ on phased genotype data derived from both microarrays and sequencing. The obtained *CYP2C19* star allele assignments were then systematically compared with the calls produced by the UT-tool and pb-StarPhase to assess general concordance. All concordance analyses and comparative evaluations were performed using R (version 4.4.3)^57^, with custom scripts developed to calculate match rates.

### Post-recruitment long-read sequencing of participants

A comprehensive long-read sequencing approach enables high-confidence characterisation of both SNVs and structural variants in *CYP2C19* and *CYP2D6*, particularly for complex or rare haplotypes that are not reliably resolved by short-read sequencing. To resolve hybrid gene variants (e.g., *CYP2D6-CYP2D7* hybrids), confirm suspected structural rearrangements, and refine star allele configurations, we performed long-read sequencing on 112 study participants. Sequencing was not conducted for two individuals (star allele diplotypes are *1/*1 for both genes based on the UT-tool, one individual has a *CYP2C19* deletion predicted from genotyping data using PennCNV).

Genomic DNA extracted from peripheral blood samples was sequenced using PacBio Revio sequencing technology to generate highly accurate circular consensus HiFi (High-Fidelity) reads. Library preparation and sequencing were performed according to the manufacturer’s standard protocols. All samples (*n* = 112) were sequenced at an aimed coverage of 20X (mean = 23.7, median = 21.7). For each sample, we required a minimum of 57.5 Gbs of raw unmapped sequence to be processed further. HiFi reads were aligned to the human reference genome (GRCh38/hg38) using pbmm2 (v1.17.0). SNVs and small insertions/deletions (indels) were called using DeepVariant (v1.6.1) with the PacBio HiFi-specific model. Haplotype phasing was carried out using HiPhase^58^ (v1.4.5). SNVs and indels were functionally annotated using the Ensembl Variant Effect Predictor (VEP, v112), with plugins including dbSNP and gnomAD for functional classification and population frequency assessment. For structural variant detection, we employed sawfish (v0.12.10)^59^, a tool tailored for sensitive detection of deletions, duplications, and complex rearrangements from long-read data.

In this study, we focused on the interpretation of genetic variants specifically within the *CYP2C19* region (chr10:94,762,681–94,855,547; GRCh38), which encompasses the full coding sequence. The well-known upstream regulatory variant CYP2C19*17 (c.-806C>T, rs12248560; chr10:94,761,900) was added to the analysis. For *CYP2D6*, we only analysed the coding region chr22:42,126,499–42,130,865. All variants with PASS filter and QC > 20 were further studied. Variants that did not have a dbSNP identifier (rsID) were considered putatively novel and were further evaluated for their functional significance. This evaluation included predicted consequences such as no function or missense variants. In addition, we examined which of the detected variants are currently listed in PharmVar^30^ (*CYP2C19* and *CYP2D6* haplotype GRCh38 files, accessed 21/03/2025). We assessed the impact of variants not covered by PharmVar, specifically those predicted to be nonfunctional or missense, on metabolic ratios.

### Long-read-derived star allele calls

We assigned *CYP2C19* and *CYP2D6* star allele diplotypes using the pb-StarPhase tool (version v1.3.2)^46^, applying a minimum read support threshold of 2. The long-read-derived star allele calls were compared with previously inferred diplotypes from Illumina short-read sequencing and genotyping array data, generated during the initial recruitment phase. For cases with discordant calls or complex SVs, we inspected and validated haplotype structures manually using Integrative Genomics Viewer. Star allele diplotypes were translated into predicted metabolic phenotypes using gene-specific Clinical Pharmacogenetics Implementation Consortium/ClinPGx allele-phenotype translation tables (accessed 18/03/2025). All diplotype-phenotype visualisations and comparison with in vivo metabolic ratios were performed using the R software^57^ (version 4.4.3).

### Drug-drug interaction

Although the use of established CYP2C19 and CYP2D6 inhibitors or inducers was among the exclusion criteria during the recruitment phase, the long intervals between the first pre-dosing and second PK sampling visits (partly due to the COVID-19 pandemic) may have resulted in changes in medication use during this period. The presence of inhibitors and inducers was assessed using electronic health records of prescription medications purchased within the six months preceding the second study visit. The data on prescription drug purchases were extracted from digital drug prescription records of the Estonian Health Insurance Fund. The selection of inhibitors and inducers was based on The Drug Interaction Flockhart Table^60^, including substrates with strong, moderate and weak evidence for interactions.

To verify the use of purchased medications and to monitor potential unregistered use of the most important CYP2C19 and CYP2D6 inhibitors or inducers, primarily form the groups of centrally acting drugs, i.e. antidepressants (inhibitors) and antiepileptics (inducers), a plasma sample from each participant was analysed. This was done using a validated, multianalyte UHPLC-HRMS method applied for therapeutic drug monitoring of psychoactive drugs at Diakonhjemmet Hospital. The samples were prepared and analysed with the same instrumentation as described for the in vivo phenotyping measurements. The method identifies potential co-medications, including the enzyme-inhibiting antidepressants bupropion, fluoxetine, fluvoxamine and paroxetine, and the enzyme-inducing antiepileptics carbamazepine and phenytoin.

### Statistical modelling

The effects of genotypes on metabolic ratios were investigated using linear regression. One member per related pairs (identity by decent > 20%) was excluded, resulting in a sample size of 113 individuals. Additionally, we removed *CYP2D6* diplotypes present in only one individual. The metabolic ratios were log-transformed, and models were adjusted for covariates with significant effects (P < 0.05) identified through forward stepwise regression. The tested covariates included age, sex, BMI, weight, clinical measurements and CYP2D6 or CYP2C19 inhibitors use during the six months before the study visit. For omeprazole, the final models considered BMI and the use of CYP2C19 inhibitors six months prior to the study visit. For metoprolol, adjustments were made for the use of CYP2D6 inhibitors six months prior. Contrasts were applied to test the differences between the effects of genotypes other than normal metaboliser, defined as individuals with two normal function alleles, which serves as the reference level in the linear regression. R software^57^ (4.4.3) was used to conduct the analysis.

Genome-wide analysis of metoprolol and omeprazole log-transformed metabolic ratios was performed using long-read data with REGENIE v3.2^61^, adjusting for sex, birth year and the first two genotype principal components. Individuals with non-European ancestry were excluded (*n* = 1), leaving 111 individuals for analysis. Regional association plots for the significant loci were generated using LocusZoom^43^.

### List of abbreviations

AUC: Area Under the Curve; BMI: Body Mass Index; CYP: Cytochrome P450; CYP2C19: Cytochrome P450 family 2 subfamily C member 19; CYP2D6: Cytochrome P450 family 2 subfamily D member 6; DDI: Drug–Drug Interaction; EstBB: Estonian Biobank; GWAS: Genome-Wide Association Study; NMa: Normal Metaboliser Allele; PM: Poor Metaboliser; UM: Ultrarapid Metaboliser; RM: Rapid Metaboliser; IM: Intermediate Metaboliser; NM: Normal Metaboliser; PK: Pharmacokinetics; SNV: Single Nucleotide Variant; SV: Structural Variant; UHPLC: Ultra High-Performance Liquid Chromatography; HRMS: High-Resolution Mass Spectrometry; PharmVar: Pharmacogene Variation Consortium.

## Supplementary information

Supplementary Materials

Supplementary Data

## Data Availability

The activities of the EstBB are regulated by the Human Genes Research Act, which was adopted in 2000 specifically for the operations of the EstBB. Individual level data analysis in the EstBB was carried out under ethical approval 1.1-12/678 and 1.1-12/151 from the Estonian Committee on Bioethics and Human Research (Estonian Ministry of Social Affairs), using data according to release applications R10 and S20 from the Estonian Biobank. All data generated or analysed during this study are included in this published article and its supplementary information files. Individual-level data at EstBB can only be accessed through EstBB. More information regarding access can be found at https://genomics.ut.ee/en/content/Estonian-biobank. The GWAS summary statistics from this study will be submitted to the GWAS Catalog.

## Code availability

The underlying code for this study is not publicly available but may be made available to researchers on reasonable request from the corresponding author.
