# Supplementary material for: Pharmacokinetic recall study of Estonian Biobank participants with novel genetic variants in CYP2C19 and CYP2D6
Source: NPJ Genom Med. 2026 Jan 18;11:10. doi: 10.1038/s41525-025-00549-6 (PMC12859152; doi:10.1038/s41525-025-00549-6)
Supplement: Supplementary file 1 — Supplementary Materials [file 41525_2025_549_MOESM1_ESM.docx]

**Supplementary Materials**

**Pharmacokinetic recall study of Estonian Biobank participants with novel genetic variants in *CYP2C19* and *CYP2D6***

**Authors:**

Kristi Krebs^1*^, Laura Birgit Luitva^1,2*^, Anette Caroline Kõre^3^, Raul Kokasaar^4#^, Maarja Jõeloo^1^, Georgi Hudjashov^1^, Kadri Maal^1^, Elisabet Størset^5,6^, Birgit Malene Wollmann^5^, Liis Karo-Astover^1^, Krista Fischer^1,2^, Estonian Biobank Research Team^1^, Volker M Lauschke^7,8,9,10^, Magnus Ingelman-Sundberg^7^, Espen Molden^5,6^, Alar Irs^3^, Kersti Oselin^4^, Jana Lass^1,3,11‡^ and Lili Milani^1‡^

1. Estonian Genome Centre, Institute of Genomics, University of Tartu, Tartu, Estonia
2. Institute of Mathematics and Statistics, University of Tartu, Tartu, Estonia
3. Tartu University Hospital, Tartu, Estonia
4. Clinic of Oncology and Haematology, North Estonia Medical Center, Tallinn, Estonia
5. Center for Psychopharmacology, Diakonhjemmet Hospital, Oslo, Norway
6. Department of Pharmacy, University of Oslo, Oslo, Norway
7. Department of Physiology and Pharmacology, Karolinska Institutet, Stockholm, Sweden
8. Dr Margarete Fischer-Bosch Institute of Clinical Pharmacology, Stuttgart, Germany
9. University of Tübingen, Tübingen, Germany
10. Department of Pharmacy, the Second Xiangya Hospital, Central South University, Changsha, China
11. Institute of Pharmacy, University of Tartu, Tartu, Estonia

* These authors contributed equally as first authors.

‡ These authors contributed equally as last authors.

# Current affiliation: West Tallinn Central Hospital, Tallinn, Estonia

Corresponding author E-mail: lili.milani@ut.ee (LM)

Banner author contributors

Estonian Biobank Research Team: Andres Metspalu, Lili Milani, Tõnu Esko, Reedik Mägi, Mait Metspalu, Mari Nelis and Georgi Hudjashov

Table of Contents

[Supplementary 3](#_Toc214632531)

[*CYP2C19* Deletion Frequency in the Estonian Biobank 3](#_Toc214632532)

[Star Allele Diplotype Assignment and Concordance Analysis 3](#_Toc214632533)

[Genome-wide screen highlights *CYP2C19* and *CYP2D6* primary contribution 4](#_Toc214632534)

[References for the supplementary 5](#_Toc214632535)

[Supplementary Figures 6](#_Toc214632536)

[Supplementary Figure 1. Study enrolment flow and the characteristics of final participants. 6](#_Toc214632537)

[Supplementary Figure 2. Probe-drug metabolic ratios across star allele diplotypes for 7](#_Toc214632538)

[Supplementary Figure 3. Concordance of *CYP2D6* star allele calls across four tools. 8](#_Toc214632539)

[Supplementary Figure 4. Concordance of *CYP2C19* star allele calls across three tools 9](#_Toc214632540)

[Supplementary Figure 5. Probe-drug metabolic ratios across metaboliser phenotypes for 10](#_Toc214632541)

[Supplementary Figure 6. Individuals with novel *CYP2D6* variants by star allele genotype and metabolic ratio. 11](#_Toc214632542)

[Supplementary Figure 7. Genome-wide association analysis of omeprazole and metoprolol metabolic ratios. 12](#_Toc214632543)

[Overview and description of Supplementary Data Tables 13](#_Toc214632544)

# Supplementary Information

## *CYP2C19* Deletion Frequency in the Estonian Biobank

Out of a total of 211,299 individuals with genotyping data, 3,859 had a deletion, while 204,393 did not. We excluded 3,047 individuals where the CNV status could not be reliably determined due to ambiguous signals. Among the 208,252 individuals with definitive CNV calls, the estimated frequency of the *CYP2C19*37* partial deletion was 1.9%, suggesting that this structural variant is more prevalent in the Estonian population than previously recognised^1^.

## Star Allele Diplotype Assignment and Concordance Analysis

Diplotypes assigned with the pb-StarPhase algorithm^2^ were used for all downstream analyses. To assess the similarities of *CYP2D6* diplotypes derived from short-read sequencing data, we compared the diplotype calls obtained using the Cyrius^3^ and Aldy^4^ tools against the results from pb-StarPhase in a subset of 43 participants (with available genome sequencing data). Both short-read-based tools performed well, with Cyrius demonstrating a concordance rate of 93.0% and Aldy achieving 90.7% when compared to the long-read-based pb-StarPhase calls (Supplementary Figure 2, Supplementary Data 4). The observed mismatches were primarily linked to hybrid allele classifications, highlighting the advantage of pb-StarPhase’s long-read approach in resolving structural complexities. The previously used UT-tool^5^ exhibited a lower concordance rate of 83.7%, largely due to its limitations in identifying hybrid alleles.

For the *CYP2C19* gene, we also compared the pb-StarPhase results with diplotype calls generated using the UT-tool and the PharmCAT^6^ algorithm across all 114 participants (GS+microarray). The overall concordance was notably low, primarily because neither the UT-tool nor PharmCAT could call structural variants for *CYP2C19*. In particular, the partial gene deletion allele *CYP2C19*37* was uniquely identified by pb-StarPhase, leading to significant mismatches with both the UT-tool (concordance: 28.1%) and PharmCAT (concordance: 41.2%, as shown in Supplementary Figure 3, Supplementary Data 4). Furthermore, the more recently characterised *CYP2C19***38* allele was not included in UT-tool’s original allele database, which contributed considerably to its lower concordance rate.

## Genome-wide screen highlights *CYP2C19* and *CYP2D6* primary contribution

To assess whether genomic regions beyond *CYP2C19* and *CYP2D6* affect the variability of drug metabolism, we conducted genome-wide association analyses (GWAS) using the metabolic ratios of omeprazole and metoprolol.

For omeprazole, the GWAS revealed a genome-wide significant peak on chromosome 10, overlapping with the *CYP2C19* locus (Supplementary Figure 7, Supplementary Data 11). The lead variant is in the *CYP2C* locus (rs71482318, P=1.7×10^-11^), consistent with prior knowledge that CYP2C19 is the primary enzyme responsible for omeprazole metabolism. This intronic lead variant is not part of any star allele but is in complete linkage disequilibrium (LD) (r^2^=1) with rs12769205 and rs4244285, which define the poor metabolizer *CYP2C19*2* allele. No other loci neared genome-wide significance (P < 5 × 10^-8^), indicating no strong evidence for other genomic regions contributing to omeprazole metabolic variability in this dataset. To assess whether the observed association was specifically driven by the *CYP2C19*2* allele, we performed a conditional analysis including rs4244285 as a covariate (in LD with rs12769205). As expected, the chromosome 10 signal at the *CYP2C19* locus was markedly attenuated (data not shown), confirming that the association was largely attributable to the *CYP2C19*2*-defining variants.

For metoprolol, the GWAS identified a prominent association peak on chromosome 22, next to the *CYP2D6* locus (Supplementary Figure 7, Supplementary Data 11). Six lead variants with the same p-value (P=6.7x10^-8^) are in *WBP2NL* or *SEPTIN3* that are adjacent to the *CYP2D6* gene. In addition, two genome-wide significant loci were identified (Supplementary Figure 7): one on chromosome 10, with the lead variant in *LRMDA* (rs182557066, P=9.5x10^-9^) and another on chromosome seven, with the lead variant in *AKAP9* (rs188755628, P=1.0x10^-8^). Both genes have been reported previously to be linked most significantly to atrial fibrillation and flutter in pooled biobank GWAS (mvp-ukbb.finngen.fi: P*_LRMDA_*=7.9x10^-22^ and P*_AKAP9_*=1.1x10^-11^). To determine whether the chromosome 22 signal was driven by known functional variants of *CYP2D6*, we performed a conditional analysis including rs3892097, the splice site variant that defines the loss-of-function allele *CYP2D6*4*, as a covariate. After conditioning, the association signal on chromosome 22, as well as the ones on chromosomes 10 and 7, were fully attenuated (data not shown), indicating that the observed signal is largely attributable to the *CYP2D6*4* allele. These findings reaffirm the central role of *CYP2D6* in metoprolol metabolism, consistent with previous results^7^.

# References for the supplementary

1. Botton, M. R. *et al.* Structural variation at the CYP2C locus: Characterization of deletion and duplication alleles. *Hum. Mutat.* **40**, e37–e51 (2019).

2. Holt, J. M. *et al.* StarPhase: Comprehensive Phase-Aware Pharmacogenomic Diplotyper for Long-Read Sequencing Data. 2024.12.10.627527 Preprint at https://doi.org/10.1101/2024.12.10.627527 (2024).

3. Chen, X. *et al.* Cyrius: accurate CYP2D6 genotyping using whole-genome sequencing data. *Pharmacogenomics J.* **21**, 251–261 (2021).

4. Hari, A. *et al.* An efficient genotyper and star-allele caller for pharmacogenomics. *Genome Res.* **33**, 61–70 (2023).

5. Reisberg, S. *et al.* Translating genotype data of 44,000 biobank participants into clinical pharmacogenetic recommendations: challenges and solutions. *Genet. Med. Off. J. Am. Coll. Med. Genet.* **21**, 1345–1354 (2019).

6. Sangkuhl, K. *et al.* Pharmacogenomics Clinical Annotation Tool (PharmCAT). *Clin. Pharmacol. Ther.* **107**, 203–210 (2020).

7. Laverdière, J. *et al.* Pharmacogenomic markers of metoprolol and α-OH-metoprolol concentrations: a genome-wide association study. *Pharmacogenomics* **24**, 441–448 (2023).

# Supplementary Figures


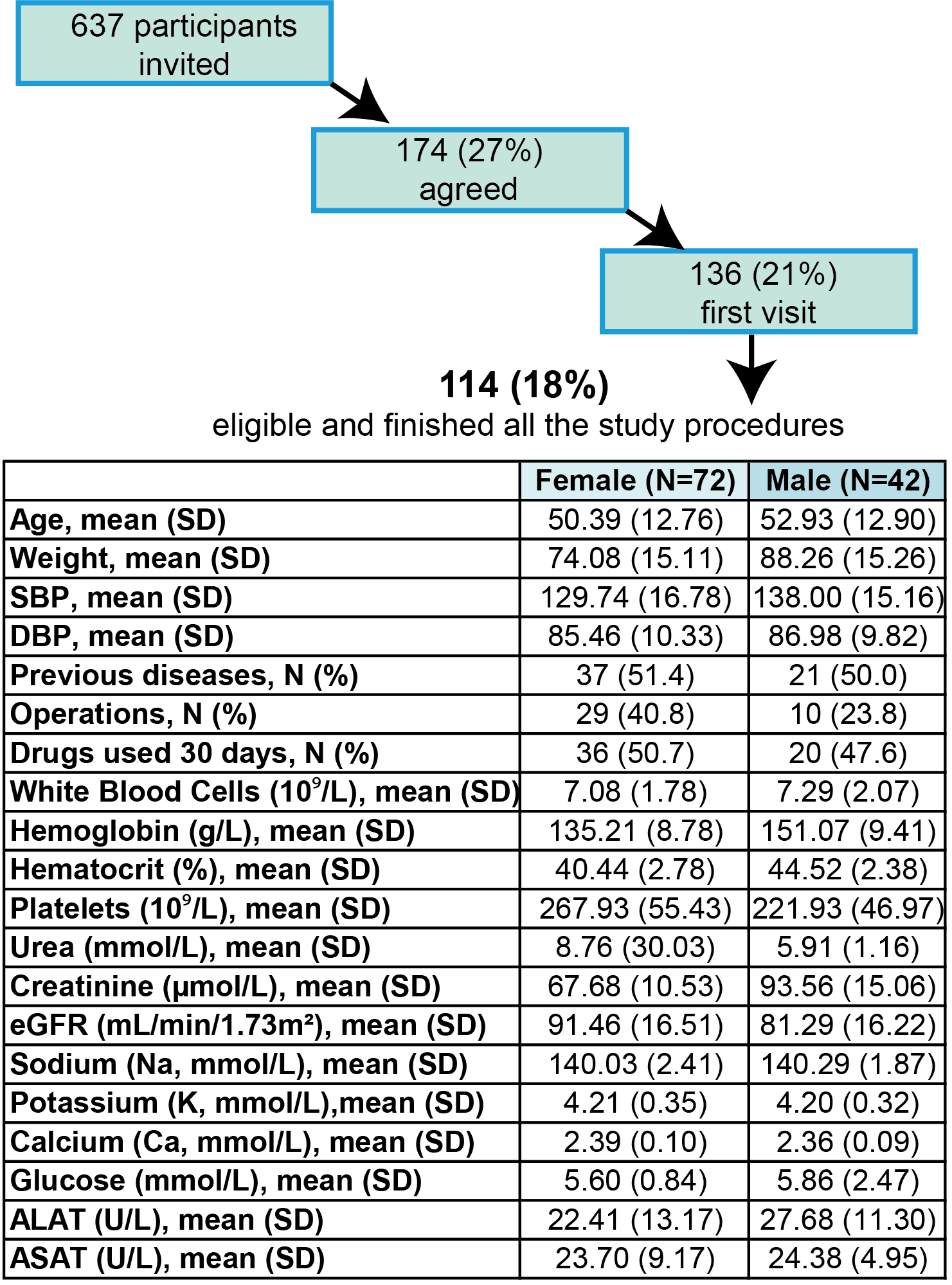


Supplementary Figure 1. Study enrolment flow and the characteristics of final participants. The table summarises the clinical characteristics of all participants who completed the study.


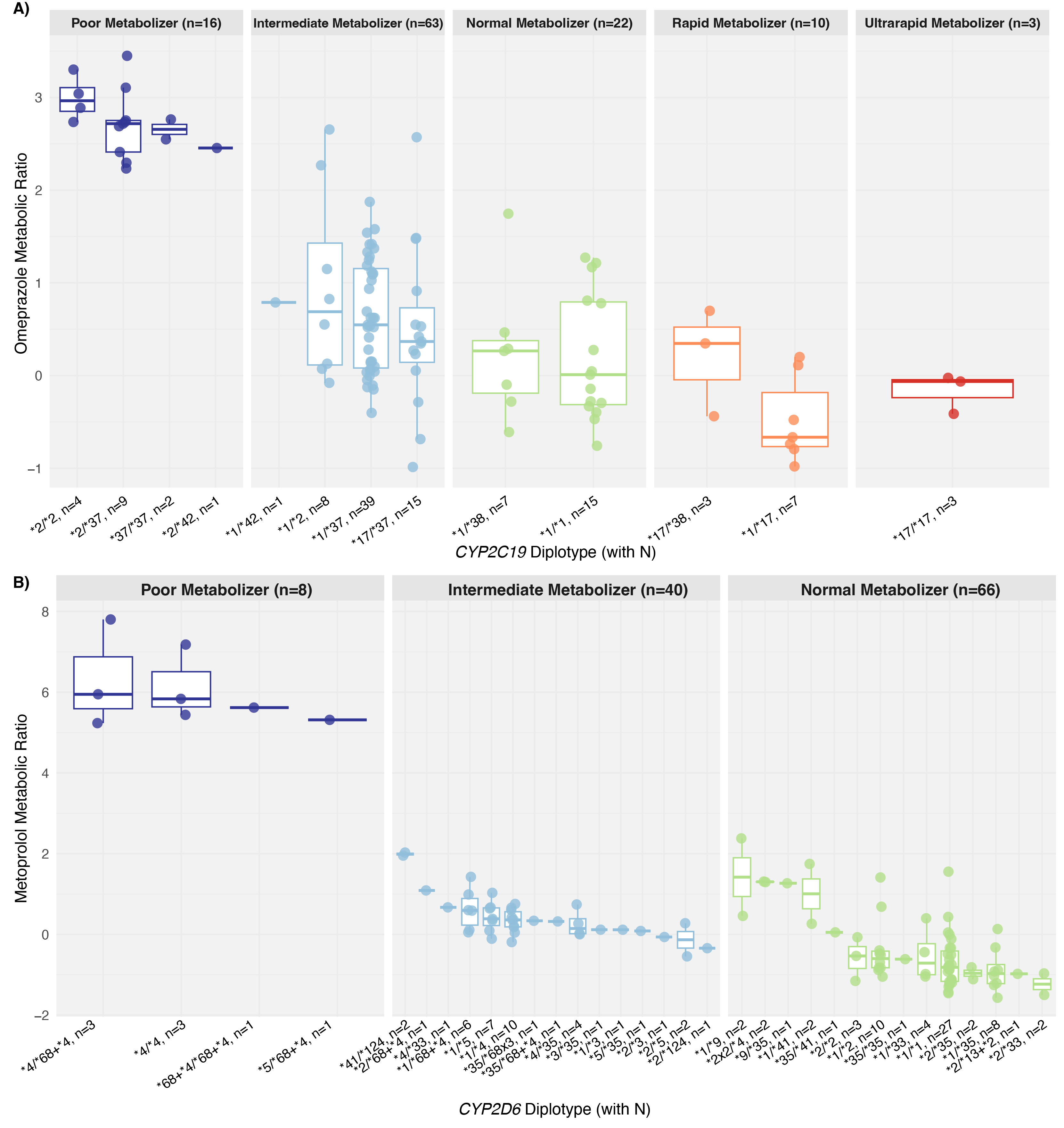


Supplementary Figure 2. Probe-drug metabolic ratios across star allele diplotypes for **(A) *CYP2C19* and (B) *CYP2D6***. The x-axis shows diplotypes (with the number of individuals per group), and the y-axis shows the metabolic ratios of omeprazole for CYP2C19 and metoprolol for CYP2D6. Genotypes are coloured by predicted metaboliser phenotype: dark blue for poor metabolisers, light blue for intermediate metabolisers, orange for rapid metabolisers, and red for ultrarapid metabolisers.


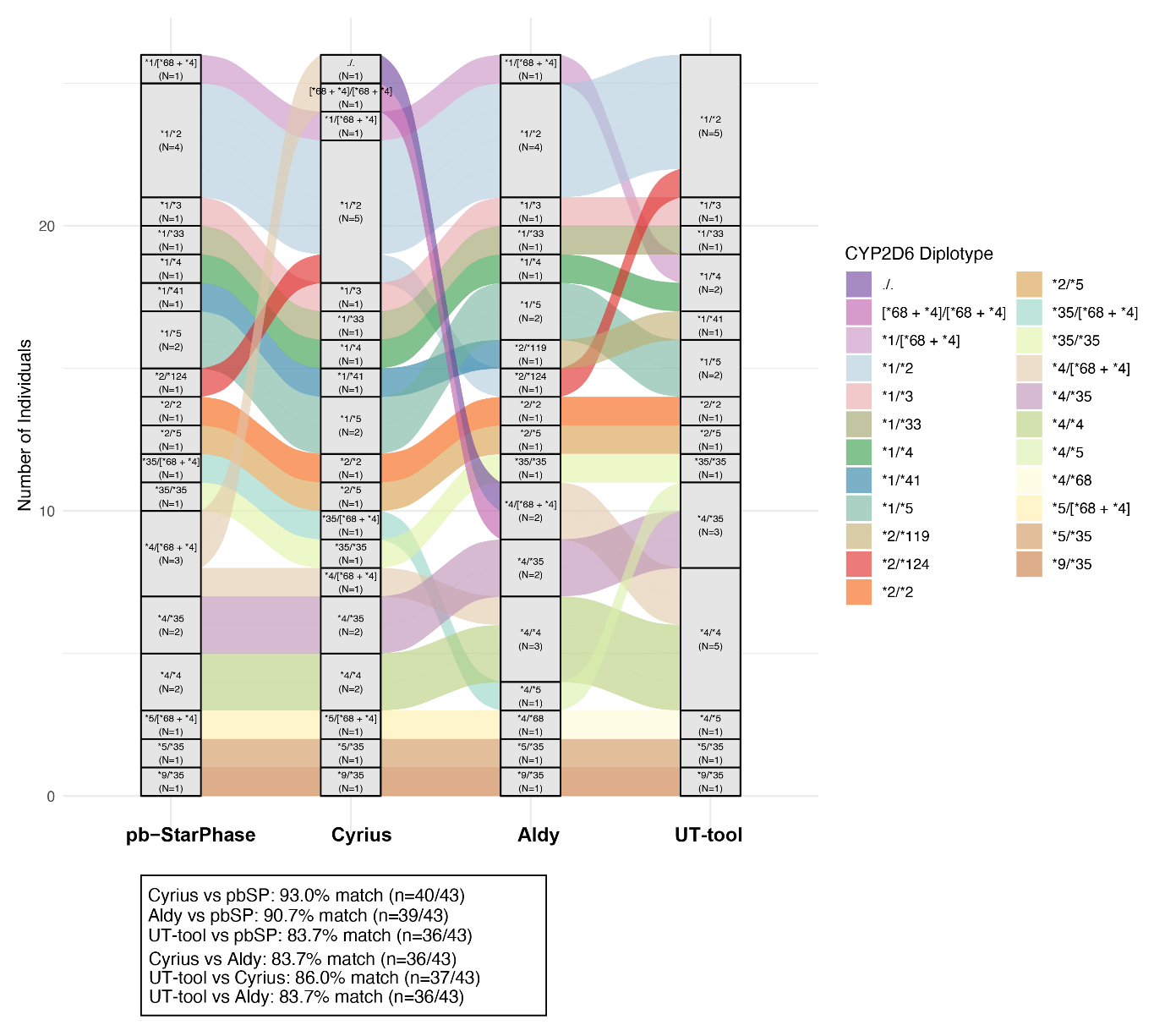


Supplementary Figure 3. Concordance of *CYP2D6* star allele calls across four tools. An alluvial plot showing differences in star allele diplotype calls in a subset of 43 participants with short-read genome sequencing data, comparing PacBio StarPhase (pb-StarPhase), Cyrius, Aldy, and the UT-tool. The y-axis indicates the number of individuals, and different colours represent diplotypes. The width of the connecting lines reflects the number of individuals. For clarity, matching *CYP2D6*1/*1* calls were excluded from the plot.


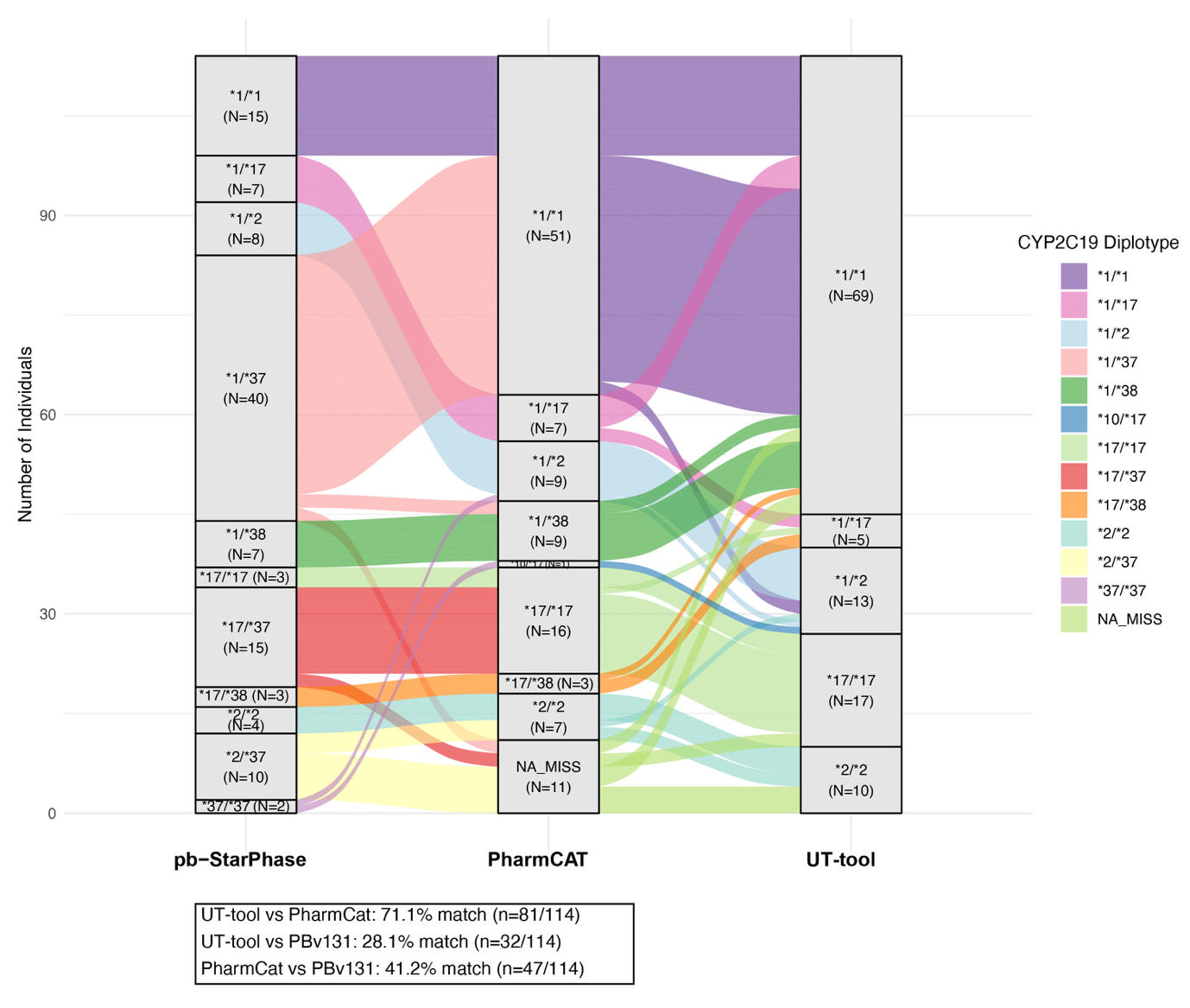


Supplementary Figure 4. Concordance of *CYP2C19* star allele calls across three tools**.** An alluvial plot showing how star allele diplotype calls (n = 114) vary between PacBio StarPhase (pb-StarPhase), PharmCAT, and UT-tool. The y-axis indicates the number of individuals, and different colours represent diplotypes. The width of the connecting lines reflects the number of individuals shared between categories.


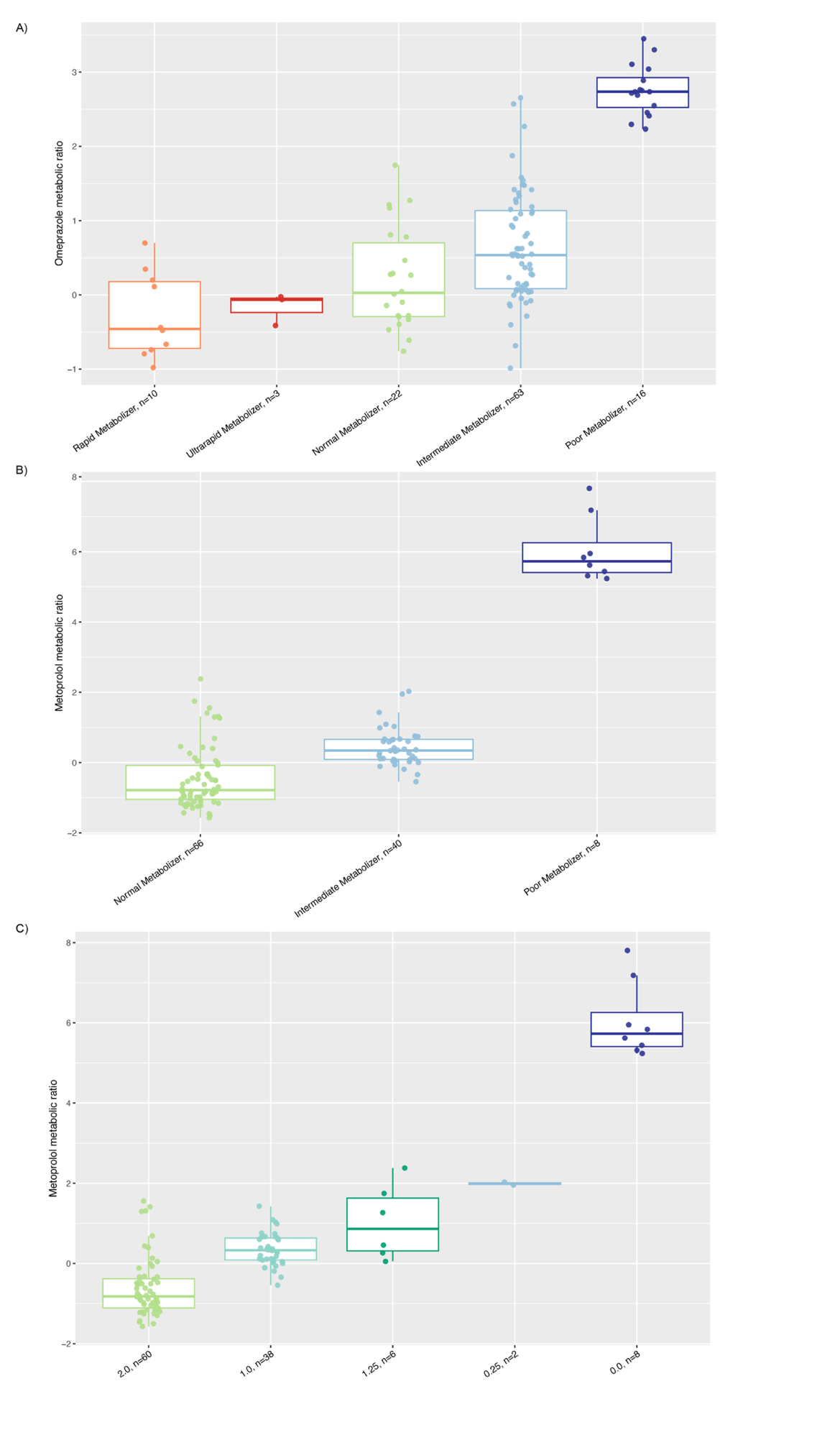


Supplementary Figure 5. Probe-drug metabolic ratios across metaboliser phenotypes for **(A) CYP2C19 and (B) CYP2D6**. The x-axis groups individuals by predicted metaboliser phenotype (A, B) and CYP2D6 activity score (C). The y-axis shows metabolic ratios of omeprazole (A) and metoprolol (B). Colour coding corresponds to phenotype: dark blue for poor metabolisers, light blue for intermediate metabolisers, orange for rapid metabolisers, and red for ultrarapid metabolisers.


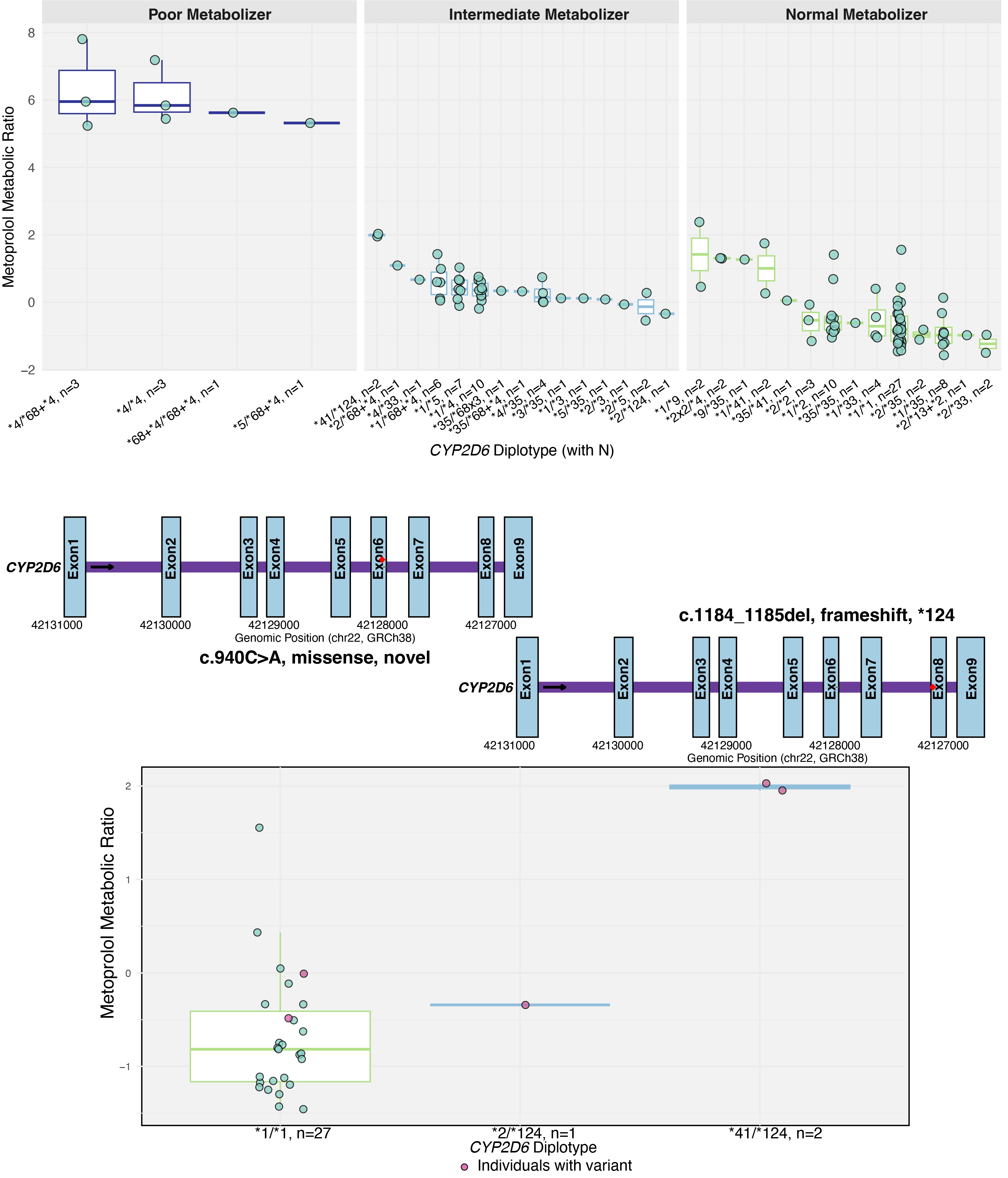


Supplementary Figure 6. Individuals with novel *CYP2D6* variants by star allele genotype and metabolic ratio. Individuals with variants are shown as pink dots; individuals without variants are shown as blue dots. The x-axis indicates diplotypes and the number of individuals per group, while the y-axis shows metoprolol metabolic ratio. Text labels on dots indicate the variant positions and predicted functional consequences. Furthermore, a schematic representation of the *CYP2D6* gene, with the exons highlighted in blue (coordinates from Ensembl), illustrates the locations of the novel missense variant and the *CYP2D6*124* nonfunctional variant.

**
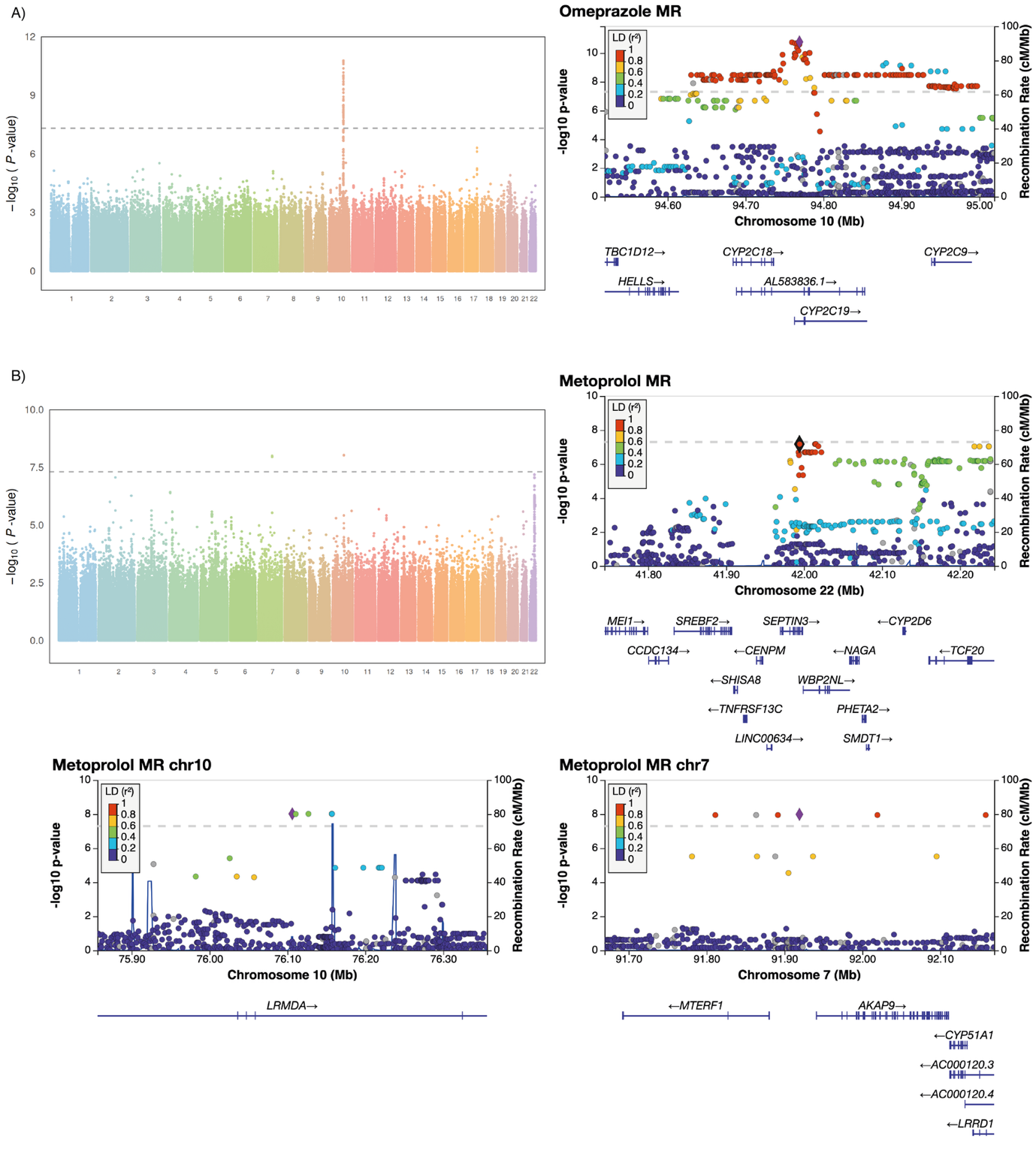
**

Supplementary Figure 7. Genome-wide association analysis of omeprazole and metoprolol metabolic ratios. Manhattan plots for omeprazole (A) and metoprolol (B) metabolic ratios are shown on the left. The y-axis represents −log10(P) for the association of SNVs, the horizontal dashed line indicates the genome-wide significance threshold (P<5×10^−8^). The genomic inflation factor (λ) was 1.0 for omeprazole (A) and 0.9 for metoprolol (B). On the right (and below for metoprolol), corresponding regional association plots display the genomic loci surrounding the top-associated variants. The x-axis shows the genomic position (in megabases, Mb), and the y-axis indicates statistical significance (−log₁₀(P)). The purple diamond marks the most significantly associated SNP within each locus. SNPs are colour-coded according to their linkage disequilibrium (LD, r²) with the lead SNP, based on data from the European population in the 1000 Genomes Project.

# Overview and description of Supplementary Data Tables

Supplementary Data 1. Rare, novel, and incompletely characterised variants in *CYP2C19* and *CYP2D6* selected for recall invitations.

Supplementary Data 2.  Inclusion criteria for study invitations and participant recruitment summary.

Supplementary Data 3. Star allele calls for the 114 study participants: pre-recruitment and post-recruitment tools.

Supplementary Data 4.  Concordance of star allele calls between different tools for *CYP2C19* and *CYP2D6*.

Supplementary Data 5.  Linear regression results assessing the effects of *CYP2C19* and *CYP2D6* phenotypes on omeprazole and metoprolol metabolic ratios, respectively.

Supplementary Data 6.  All variants detected by long-read sequencing (n = 112) within the *CYP2C19* gene (including the *CYP2C19*17* regulatory variant).

Supplementary Data 7. Linear regression results assessing *CYP2C19* diplotype effects on omeprazole metabolic ratios

Supplementary Data 8.  All variants detected by long-read sequencing (n = 112) within the *CYP2D6* gene

Supplementary Data 9.  Linear regression results assessing *CYP2D6* diplotype effects on metoprolol metabolic ratios

Supplementary Data 10. Presence of CYP2D6 and CYP2C19 inhibitors among study participants to assess the impact of drug–drug interactions.

Supplementary Data 11. Genome-wide association results of log-transformed metabolic ratios of omeprazole and metoprolol
